# Supplementary material for: The Synergistic Mechanism of Total Saponins and Flavonoids in Notoginseng–Safflower against Myocardial Infarction Using a Comprehensive Metabolomics Strategy
Source: Molecules. 2022 Dec 13;27(24):8860. doi: 10.3390/molecules27248860 (PMC9782856; doi:10.3390/molecules27248860)
Supplement: Supplementary file 1 [file molecules-27-08860-s001.zip › molecules-2049622-supplementary.pdf]

Supporting information for:

The synergistic mechanism of total saponins and flavonoids in Notoginseng-Safflower against myocardial infarction by a comprehensive metabolomics strategy

Meng Fang[1,†], Yu-Qing Meng[1,†], Zhi-Yong Du[1], Meng-Qiu Guo[1], Yong Jiang[1], Peng-Fei Tu[1], Kun Hua[2,*], Ying-Yuan Lu[1,*], Xiao-Yu Guo[1,*]

1 State Key Laboratory of Natural and Biomimetic Drugs, School of Pharmaceutical Sciences, Peking University, Beijing 100191, China

2 Department of Cardiovascular Surgery, Beijing Anzhen Hospital, Capital Medical University, Beijing 100029, China

* Correspondence: kunhua@mail.ccmu.edu.cn (K.H.); luyingyuan518@bjmu.edu.cn (Y.Y.L.); guox-iaoyu@bjmu.edu.cn (X.Y.G.)

† These authors contributed equally to this study.

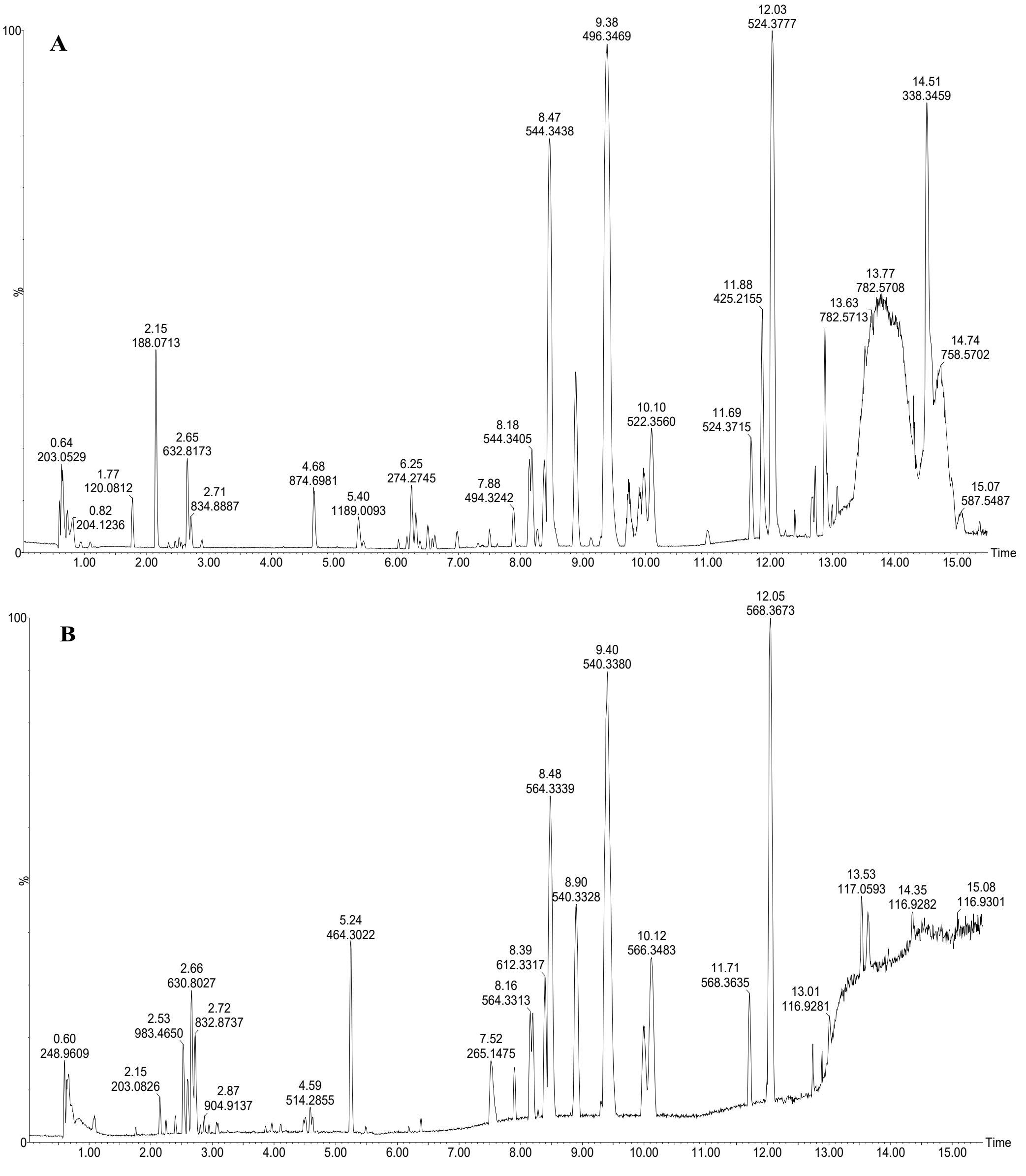

Figure S1 The representative base peak intensity chromatograms of the rat plasma samples in ESI positive and negative mode by UPLC-QTOF/MS. A, positive mode; B, negative mode.

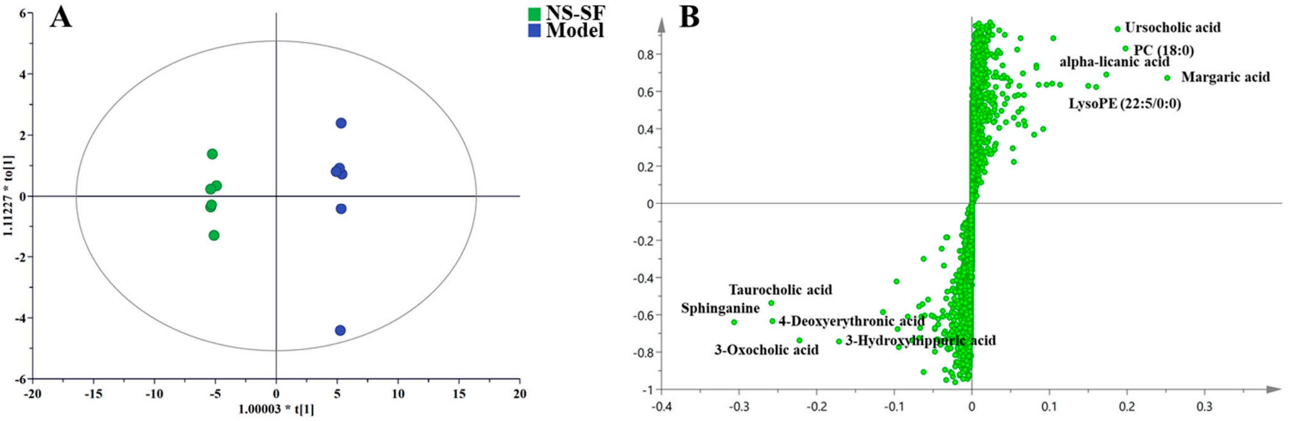

Figure S2 The OPLS-DA analysis of the plasma samples from NS-SF and model rats by UPLC-QTOF/MS analysis. A, OPLS-DA score plot of plasma samples; B, *S*-plot from OPLS-DA of plasma samples.

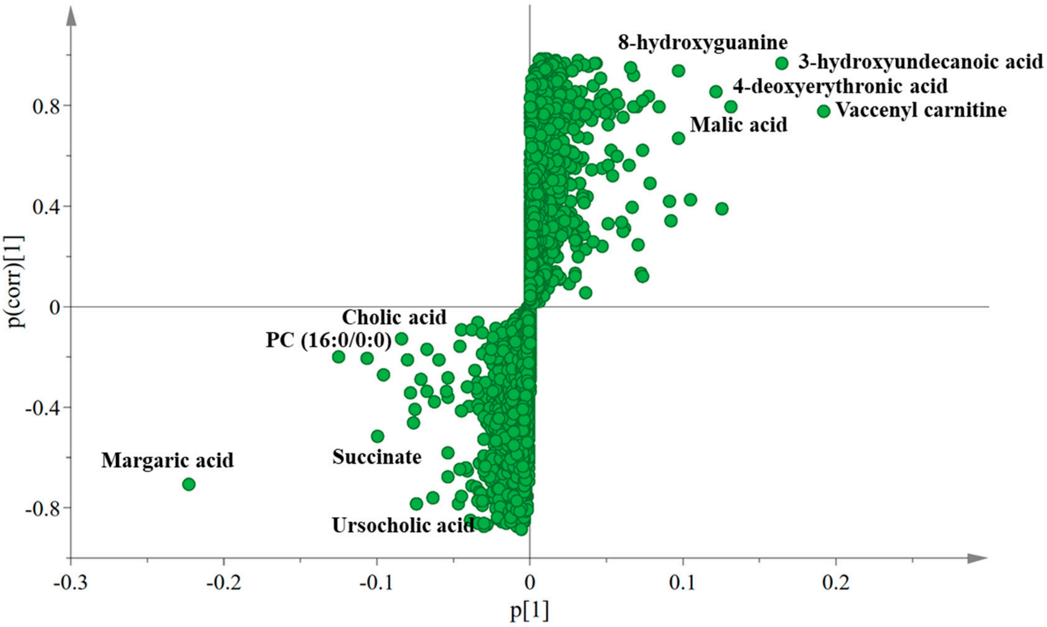

Figure S3 *S*-plot of the plasma samples from sham and model rats by UPLC-QTOF/MS analysis.
